# Supplementary material for: Degradation of 5-Dialkylamino-Substituted Chlorsulfuron Derivatives in Alkaline Soil
Source: Molecules. 2022 Feb 23;27(5):1486. doi: 10.3390/molecules27051486 (PMC8911686; doi:10.3390/molecules27051486)
Supplement: Supplementary file 1 [file molecules-27-01486-s001.zip › Crop safety assay (revised).pdf]

Crop safety[1-2]

Wheat safety test

The wheat was dealt with selected compound through pre- and post-emergence treatments via pot trials in greenhouse (25 ± 2 °C). The applied concentration was 30 and 60 $g \cdot ha^{-1}$, and each treatment was triplicated. For pre-emergence treatment, the compound was sprayed onto the soil in cups (d=7 cm) following the seeding performance. After that, the moisture was maintained and cultivated in greenhouse. The fresh weight of the cover crop was measured after 22 days, and the safety was represented by the inhibition rate. The variance analysis of the data was performed by the Duncan multiple comparisons with the SPSS 22.0 software.

For post-emergence treatment, the seeding was conducted following adjusting the moisture and temperature until the wheat grew to 4-leaf stage. The compound was sprayed, and the result was measured after 28 days, the same procedure as pre emergence treatment.

Corn safety test

The corn safety was conducted through pre- and post-emergence treatments as wheat. After pre-emergence treatment of the relevant concentration of selected compound in cups (d=12 cm), the seeding was performed following the moisture and temperature adjustment. The result was determined after 16 days, and variance analysis was proceeded as wheat safety.

For post emergence treatment, the seeding was conducted until the corn grew to 3-leaf stage. The compound was sprayed, and the result was measured after 23 days, the same procedure as pre-emergence treatment.

The data analysis of variance was completed by Duncan multiple comparison using SPSS 22.0, a statistical analysis software for analysis of variance, regression analysis and correlation analysis etc.

References

1.Zhou, S.; Meng, F. F.; Hua, X. W.; Li, Y. H.; Liu, B.; Wang, B. L.; Chen, J.; Chen, A. L.; Li, Z. M., Research on Controllable Degradation of Novel Sulfonylurea Herbicides in Acidic and Alkaline Soils. *J. Agric. Food Chem.* **2020**, 68 (10), 3017-3025.

2.Meng, F. F.; Wu, L.; Gu, Y. C.; Zhou, S.; Li, Y. H.; Chen, M. G.; Zhou, S.; Zhao, Y. Y.; Ma, Y.; Li, Z. M. Research on the Controllable Degradation of N-methylamido and Dialkylamino Substituted at the 5th Position of the Benzene Ring in Chlorsulfuron in Acidic Soil. *RSC Adv.* **2020**, 10 (30), 17870-17880.
