# Supplementary material for: Degradation of 5-Dialkylamino-Substituted Chlorsulfuron Derivatives in Alkaline Soil
Source: Molecules. 2022 Feb 23;27(5):1486. doi: 10.3390/molecules27051486 (PMC8911686; doi:10.3390/molecules27051486)
Supplement: Supplementary file 1 [file molecules-27-01486-s001.zip › Herbicidal Activity of Compounds NL101-NL108.pdf]

Biological assay

Pot trials

Pot trials for herbicidal activities against *Echinochloa crusgalli*, *Digitaria sanguinalis, Amaranthus tricolor*, and *Brassica campestris* were tested through pre- and post-emergence treatment at 15 $g{\cdot}ha^{-1}$ and 150 $g{\cdot}ha^{-1}$ with chlorsulfuron as a positive control in greenhouse (25 ± 2 °C) following reported procedures.[1-2] And each treatment was triplicated. Pre-emergence treatment: sandy clay (100 g) was placed into a 6 cm-diameter plastic flowerpot and 15 sprouting weed seeds were planted (about 0.6 cm depth) and sprayed of tested chemicals with spray tower at 900$L{\cdot}ha^{-1}$ volume. Post-emergence treatment: the seeding was conducted following adjusting the moisture and temperature, and the tested chemicals were sprayed until the weeds grew up to one leaf and one stem stage. For pot trials, the fresh weight of weeds was determined after 21 days, and the inhibition rate was calculated. The variance analysis of the data was performed by the Duncan multiple comparisons with the DPS16.5 software.[3]

The herbicidal activity of target compounds NL101-NL108 was reported in our previous study.[4]

Table S3. The Herbicidal Activity of Target Compounds NL101-NL108.

Compound	Concentration ($g{\cdot}ha^{-1}$)	Herbicidal activity (%)							
		Brassica campestris		*Amaranthus tricolor*		*Echinochloa crusgalli*		*Digitaria sanguinalis*	
		Pre	*Post*	*Pre*	*Post*	*Pre*	*Post*	*Pre*	*Post*
**Chlosulfuron**	15	93.7	87.5	97.4	99.7	72.6	71.8	8.1	44.8
	150	97.9	92.3	98.8	99.8	89.3	89.5	30.1	48.3
**NL101**	15	74.6	85.8	69.6	97.0	77.9	85.8	74.3	15.0
	150	88.5	92.1	92.4	100	87.8	88.0	93.2	36.2
**NL102**	15	87.9	92.9	82.9	91.0	73.5	89.4	24.3	16.3
	150	91.5	98.1	92.4	100	81.2	89.7	75.7	43.6
**NL103**	15	79.5	67.5	69.6	96.0	71.3	72.3	48.6	13.8
	150	95.8	73.1	92.4	99.5	76.8	78.6	60.8	35.2
**NL104**	15	57.7	87.3	44.9	95.0	56.9	81.4	51.4	15.4
	150	87.9	91	79.1	97.0	79.0	85.2	64.9	26.7
**NL105**	15	65.8	43.2	97.2	24.9	74.1	40.8	37.8	0
	150	95.8	88.9	99.1	47.9	89.1	55.4	52.8	20.7
**NL106**	15	81.9	94.7	77.2	100	72.4	83.2	58.1	11.4
	150	84.3	96.3	90.5	100	83.4	84.8	71.6	21.8
**NL107**	15	64.3	75.3	84.8	0	10.3	55.9	0	0
	150	94.6	93.8	98.1	9.0	86.2	68.0	9.8	18.6
**NL108**	15	41.0	34.6	92.1	0	0	15.4	0	0
	150	94.6	88.9	97.2	0	81.0	67.8	0	4.7

References

1.Wang, B. L.; Dμggleby, R. G.; Li, Z. M.; Wang, J. G.; Li, Y. H.; Wang, S. H.; Song, H. B. Synthesis, crystal structure and herbicidal activity of mimics of intermediates of the KARI reaction. *Pest Manage. Sci.* **2005**, 61, 407–412.

2.Teaney, S. R.; Armstrong, L.; Bentley, K.; Cotterman, D.; Leep, D.; Liang, P. H.; Powley, C.; Summers, J.; Cranwell, S.; Lichtner, F.; Stichbury, R. DPX-KE459 A new sulfonylurea for postemergence grass and broadleaf weed control in cereals. *Brighton Crop Prot. Conf. Weeds.* **1995**, 1, 49.

3.Zhou, S.; Meng, F. F.; Hua, X. W.; Li, Y. H.; Liu, B.; Wang, B. L.; Chen, J.; Chen, A. L.; Li, Z. M., Research on Controllable Degradation of Novel Sulfonylurea Herbicides in Acidic and Alkaline Soils. *J. Agric. Food Chem.* **2020**, 68 (10), 3017-3025.

4.Meng, F. F.; Wu, L.; Gu, Y. C.; Zhou, S.; Li, Y. H.; Chen, M. G.; Zhou, S.; Zhao, Y. Y.; Ma, Y.; Li, Z. M. Research on the Controllable Degradation of N-methylamido and Dialkylamino Substituted at the 5th Position of the Benzene Ring in Chlorsulfuron in Acidic Soil. *RSC Adv.* **2020**, 10 (30), 17870-17880.
