# Supplementary material for: Degradation of 5-Dialkylamino-Substituted Chlorsulfuron Derivatives in Alkaline Soil
Source: Molecules. 2022 Feb 23;27(5):1486. doi: 10.3390/molecules27051486 (PMC8911686; doi:10.3390/molecules27051486)
Supplement: Supplementary file 1 [file molecules-27-01486-s001.zip › Report of soil analysis in Chinese.pdf]

CMA
170012051117

监 测 报 告

报告编号：HJ-F-FX-202012-031

委 托 单 位	南开大学
委托单位地址	天津市南开区卫津路94号
监 测 内 容	土壤分析

天津市生态环境监测中心（盖章）

报告说明

1、报告无本中心报告专用章、骑缝章无效。

2、对于非本中心人员采集的样品，结果仅对客户送检样品结果负责。

3、对现场不可复现的样品，仅对采样所代表的时间和空间负责。

4、未经书面授权，不得部分复制本报告。

5、未经本中心同意，该监测报告不得用于商业性宣传。

6、因客户提供的信息有误，影响监测结果的有效性时，造成的一切后果与本中心无关。

地　　址：天津市南开区复康路19号

电　　话：022-87671699

传　　真：022-87671699

邮政编码：300191

电子邮箱：temcjcglb@tj.gov.cn

送检日期：2020年12月3日

分析日期：2020年12月07日-2020年12月08日

方法和仪器：

项目	方法及依据	仪器名称、型号和编号
pH值	土壤 pH值的测定 电位法（HJ 962-2018）	SevenExcellence S500-k pH（酸度）计(B546705501)<br>CPA225D型 电子分析天平(24190372)
有机质	土壤检测 第6部分：土壤有机质的测定 NY/T1121.6-2006	滴定管(滴定管-分-02)<br>BSA124S型 电子分析天平(29490274)
阳离子交换量	土壤 阳离子交换量的测定 三氯化六氨合钴浸提 分光光度法（HJ 889-2017）	DR6000 双光束紫外可见分光光度计(1492931)<br>OHAOS AR2140 电子天平(1201310779)
机械组成（质地）	森林土壤颗粒组成（机械组成）的测定密度计法（LY/T 1225-1999）	TM-85土壤密度计(341)<br>BSA124S型 电子分析天平(29490274)

项目及结果：

结果 项目（单位） 样品名称	pH值（无量纲）	阳离子交换量（cmol⁺/kg）	有机质（g/kg）	机械组成（质地）	样品状态描述
土1	8.39	7.3	19.4	粘壤土	褐色块状
土2	5.46	14.4	8.37	砂质壤土	褐色块状

备注：
1. 监测方法为客户指定。
2. 样品名称为客户提供信息，中心对此真实性不承担责任。

编制人：[illegible]　　审核人：[illegible]

签发人：[illegible]　　签发日期：2021年1月6日

态环境监测 ★ 骑缝章

附表

样品名称	土壤机械组成（各粒级含量）	
土1	1-2mm 粒级含量（g/kg）	7.95
	0.5-1mm 粒级含量（g/kg）	24.6
	0.25-0.5mm 粒级含量（g/kg）	23.3
	粉（砂）粒 0.05-0.02mm 粒级含量（g/kg）	79.0
	粉（砂）粒 0.02-0.002mm粒级含量（g/kg）	286
	粘粒<0.002mm粒级含量（g/kg）	282
	细砂+极细砂0.25-0.05mm粒级含量（g/kg）	297
	砂粒2.0-0.05mm粒级含量（g/kg）	353
	粉(砂)粒0.05-0.002mm粒级含量（g/kg）	365
土2	1-2mm 粒级含量（g/kg）	0.750
	0.5-1mm 粒级含量（g/kg）	3.81
	0.25-0.5mm 粒级含量（g/kg）	7.08
	粉（砂）粒 0.05-0.02mm 粒级含量（g/kg）	125
	粉（砂）粒 0.02-0.002mm粒级含量（g/kg）	179
	粘粒<0.002mm粒级含量（g/kg）	105
	细砂+极细砂0.25-0.05mm粒级含量（g/kg）	579
	砂粒2.0-0.05mm粒级含量（g/kg）	591
	粉(砂)粒0.05-0.002mm粒级含量（g/kg）	304
