# Supplementary material for: Degradation of 5-Dialkylamino-Substituted Chlorsulfuron Derivatives in Alkaline Soil
Source: Molecules. 2022 Feb 23;27(5):1486. doi: 10.3390/molecules27051486 (PMC8911686; doi:10.3390/molecules27051486)
Supplement: Supplementary file 1 [file molecules-27-01486-s001.zip › Report of soil analysis in English.pdf]

Tianjin Institude of Agriculture Resource and Environment

Analysis Report

Rwquester	Nankai University	Report number	HJ-F-FX-202012-013
Sample name	Soil samples	Date of receipt	December 3th, 2020
		Date of report	January 6th, 2021
Analysis items	Mechancal composition,pH,Organic matter,Cation exchange capacity		
Number of samples		Soil 1	Soil 2
pH		8.39	5.46
Cation exchange capacity ($cmol^{+}$/kg)		7.3	14.4
Organic matter(g/kg)		19.4	8.37
Sample status		Brown ,lupm	Brown ,lupm
Mechancal composition	Texture class(g/kg)		
Soil 1	1-2mm(g/kg)		7.95
	0.5-1mm(g/kg)		24.6
	0.25-0.5mm(g/kg)		23.3
	0.05-0.02mm(g/kg)		79.0
	0.02-0.002mm(g/kg)		286
	＜0.02mm(g/kg)		282
	0.25-0.05mm(g/kg)		297
	2.0-0.05mm(g/kg)		353
	0.05-0.002mm(g/kg)		365
Soil 2	1-2mm(g/kg)		0.750
	0.5-1mm(g/kg)		3.81
	0.25-0.5mm(g/kg)		7.08
	0.05-0.02mm(g/kg)		125
	0.02-0.002mm(g/kg)		179
	＜0.02mm(g/kg)		105
	0.25-0.05mm(g/kg)		579
	2.0-0.05mm(g/kg)		591
	0.05-0.002mm(g/kg)		304
