# Supplementary material for: Degradation of 5-Dialkylamino-Substituted Chlorsulfuron Derivatives in Alkaline Soil
Source: Molecules. 2022 Feb 23;27(5):1486. doi: 10.3390/molecules27051486 (PMC8911686; doi:10.3390/molecules27051486)
Supplement: Supplementary file 1 [file molecules-27-01486-s001.zip › Soil degradation assay (revised).pdf]

Soil degradation research was conducted by the following steps.

Soil selection: The alkaline soil was derived from the upper-layer (0–25 cm) in fresh farm land, and air-dried in the shade, shifted through 2 mm sieve according to the Chinese National Standard GB/T 31270.1-2014.[1] The soil texture, pH value, organic matter, cation exchange capacity (CEC) and particle size analysis of tested alkaline soil were determined by Tianjin Institute of Agricultural Resources and Environment Science.

HPLC conditions: The target compounds were explored and standard curves were established according to the Chinese National Standard GB/T 16631-2008.[2] Chromatographically pure methanol, acetonitrile and ultrapure water (pH 3.0) were used as the mobile phase. The retention time should remain between 10-20 minutes.

Standard curve: The standard curves used for quantitative conversion were established with the injection volume of 10 μL at 20 °C. The concentration range of the standard curve was between 200 $ug \cdot mL^{-1}$ and 0.025 $ug \cdot mL^{-1}$ at 20 °C.

Measurement of the Recovery Rate: According to the Chinese National Standard GB/T 31270.1-2014, the Chinese Agricultural Industry Standard NY/T788-2004 and the Chinese Agricultural Industry Standard NY/T788-2018, the concentration of the test compounds in 20 g of soil in a 100 mL conical flask were 5 $mg \cdot kg^{-1}$, 2 $mg \cdot kg^{-1}$, and 0.5 $mg \cdot kg^{-1}$ (adjusted with an acetonitrile solution), respectively.[1,3-4] Each concentration was repeated 5 times and recovery rate should range from 70% to 110% and guarantee the coefficient of variation <5% to ensure the reliability of the method.

The extraction method: The regulation of 60% water holding capacity (3.5 mL) after the acetonitrile evaporated completely (about 5 min) was implemented and the soil samples were mixed well. Suitable extraction solvent was added into the flask and then was shook in the thermostatic oscillator for 3 h at 200 $rpm \cdot min^{-1}$. The samples were centrifuged at 6500 rpm in a Thermo Scientific centrifuge at 20℃ for 2 min. The supernatant liquid were combined and concentrated. Then dichloromethane (30 mL× 2) and 30 mL of HPLC grade water were used for the extraction of the residues. The organic phase were combined and dried by anhydrous sodium sulfate. And then the mixture were filtered and concentrated at 25℃. The concentrated samples were dissolved into 10 mL acetonitrile and shook in oscillator at room temperature for 1 h. The solutions were filtered through millipore filter (organic, nylon-66, 0.22 μm) for HPLC analysis.

Cultivation of samples and management: Each sample with its concentration at 5 $mg \cdot kg^{-1}$ was added, 60% water holding capacity was then regulated. The sealed soil samples were cultivated in a biochemical incubator at 25±1℃ and 80% humidity in the dark. The degradation curves followed the first order kinetic equation $Ct = C_0 \times e^{-kt}$. DT_{50} were calculated according to the formula: $DT_{50} = \ln 2/k$ and the statistical analysis was also guaranteed by the triplicated data.

DT_{50} of the test compounds were calculated which is shown in Table S2.

Table S2 Kinetic Parameters for Alkaline Soil (pH 8.39) Degradation.

**compound**	**kinetic equations of soil degradation**	**correlation coefficient (R^2)**	**DT_{50} (days)**

**NL101**	C_t =4.9494e$^{-0.2285t}$	0.9688	3.03
**NL102**	C_t = 4.5826e$^{-0.1225t}$	0.9905	5.66
**NL103**	C_t = 4.5583e$^{-0.0895t}$	0.9914	7.74
**NL104**	C_t = 3.8846e$^{-0.0476t}$	0.9938	14.56
**NL105**	C_t =4.9726e$^{-0.1087t}$	0.9988	6.38
**NL106**	C_t = 5.0341e$^{-01084t}$	0.9735	6.39
**NL107**	C_t = 4.9213e$^{-0.0845t}$	0.9975	8.20
**NL108**	C_t = 5.103e$^{-0.0509t}$	0.9912	13.62
**Chlorsulfuron**	C_t = 4.304e$^{-0.0044t}$	0.9899	157.53

References

1. The Institute for the Control of Agrochemicals under the Ministry of Agriculture. *Chinese National Standard GB/T 31270.1-2014. Test Guidelines on Environmental Safety Assessment for Chemical Pesticides—Part 1: Transformation in Soils;* Oct 10, **2014**.
2. National Chemical Standardization Technical Committee. *Chinese National Standard GB/T 16631-2008. General rules for high performance liquid chromatography*; Jun 18, **2008**.
3. Ministry of Agriculture, Pesticide Testing Center. *Chinese Agricultural Industry Standard NY/T 788-2004. Guideline on pesticide residue trials*; Apr 16, **2004**.
4. Ministry of Agriculture, Pesticide Testing Center. *Chinese Agricultural Industry Standard NY/T 788-2018. Guideline on pesticide residue trials*; July 27, **2018**.
