# Supplementary material for: Degradation of 5-Dialkylamino-Substituted Chlorsulfuron Derivatives in Alkaline Soil
Source: Molecules. 2022 Feb 23;27(5):1486. doi: 10.3390/molecules27051486 (PMC8911686; doi:10.3390/molecules27051486)
Supplement: Supplementary file 1 [file molecules-27-01486-s001.zip › Table S1.pdf]

Table S1. The herbicidal activity of 5-dialkylamino-substituted compounds against both dicotyledons and monocotyledons.

Compound	Concentration (g·ha $^{-1}$)	Herbicidal activity (%)							
		Brassica campestris		*Amaranthus tricolor*		*Echinochloa crusgalli*		*Digitaria sanguinalis*	
		Pre	*Post*	*Pre*	*Post*	*Pre*	*Post*	*Pre*	*Post*
**Chlosulfuron**	15	93.7	87.5	97.4	99.7	72.6	71.8	8.1	44.8
**NL101**	15	74.6	85.8	69.6	97.0	77.9	85.8	74.3	15.0
**NL102**	15	87.9	92.9	82.9	91.0	73.5	89.4	24.3	16.3
**NL103**	15	79.5	67.5	69.6	96.0	71.3	72.3	48.6	13.8
**NL104**	15	57.7	87.3	44.9	95.0	56.9	81.4	51.4	15.4
**NL105**	15	65.8	43.2	97.2	24.9	74.1	40.8	37.8	0
**NL106**	15	81.9	94.7	77.2	100	72.4	83.2	58.1	11.4
**NL107**	15	64.3	75.3	84.8	0	10.3	55.9	0	0
**NL108**	15	41.0	34.6	92.1	0	0	15.4	0	0
